# Analysis of *In-Vivo* LacR-Mediated Gene Repression Based on the Mechanics of DNA Looping

**DOI:** 10.1371/journal.pone.0000136

**Published:** 2006-12-27

**Authors:** Yongli Zhang, Abbye E. McEwen, Donald M. Crothers, Stephen D. Levene

**Affiliations:** 1 Departments of Chemistry and Molecular Biophysics and Biochemistry, Yale University, New Haven, Connecticut, United States of America; 2 Institute of Biomedical Sciences and Technology, University of Texas at Dallas, Richardson, Texas, United States of America; 3 Department of Molecular and Cell Biology, University of Texas at Dallas, Richardson, Texas, United States of America; The Babraham Institute, United Kingdom

## Abstract

Interactions of *E. coli lac* repressor (LacR) with a pair of operator sites on the same DNA molecule can lead to the formation of looped nucleoprotein complexes both *in vitro* and *in vivo*. As a major paradigm for loop-mediated gene regulation, parameters such as operator affinity and spacing, repressor concentration, and DNA bending induced by specific or non-specific DNA-binding proteins (e.g., HU), have been examined extensively. However, a complete and rigorous model that integrates all of these aspects in a systematic and quantitative treatment of experimental data has not been available. Applying our recent statistical-mechanical theory for DNA looping, we calculated repression as a function of operator spacing (58–156 bp) from first principles and obtained excellent agreement with independent sets of *in-vivo* data. The results suggest that a linear extended, as opposed to a closed v-shaped, LacR conformation is the dominant form of the tetramer *in vivo*. Moreover, loop-mediated repression in wild-type *E. coli* strains is facilitated by decreased DNA rigidity and high levels of flexibility in the LacR tetramer. In contrast, repression data for strains lacking HU gave a near-normal value of the DNA persistence length. These findings underscore the importance of both protein conformation and elasticity in the formation of small DNA loops widely observed *in vivo*, and demonstrate the utility of quantitatively analyzing gene regulation based on the mechanics of nucleoprotein complexes.

## Introduction

The *lac* operon of *E. coli* provides an important paradigm for gene regulation [1], in which DNA looping is a central aspect of transcriptional repression. The gene products of the *lac* operon are three enzymes important for metabolism of lactose, an alternative cellular energy source. In the wild-type *lac* operon there are three *lac* repressor (LacR) binding sites, or operators: one primary operator (O_1_) located at the +11 position relative to the start of transcription, and two auxiliary operators located 92 bp upstream (O_3_) and 401 bp downstream (O_2_) relative to the primary binding site. DNA looping between the primary operator and either of the auxiliary operators enhances occupancy of the primary site by LacR [2], [3], thereby blocking transcription by preventing RNA polymerase binding to the promoter.

The Record [4] and Müller-Hill [5] groups reported classic studies of repression as a function of the helical phasing or DNA length between a primary and one auxiliary *lac* operator, providing early evidence for DNA looping as a mode of transcriptional control. These results and those of studies involving other proteins [6] have led to a long-standing question: how can DNA loops shorter than 100 bp form efficiently *in vivo*, given the large energy barrier created by strong DNA bending and/or twisting deformations [7], [8]? The prevailing explanation is that a DNA molecule has greater apparent flexibility *in vivo* and thus the actual DNA bending and twisting energy for loop formation is lower than that estimated from *in-vitro* DNA-elasticity parameters. Such enhanced apparent flexibility could be attributed to nonlinear behavior of DNA elasticity accompanying strong DNA distortion [9]–[13], or result from dynamic and non-specific protein binding and bending [14]–[19]. Indeed, Becker *et al*. [19] addressed the latter hypothesis directly by investigating effects of deletion of both genes that encode subunits for the non-specific DNA-binding protein HU. They found that loop-mediated repression mediated by LacR was substantially reduced in HU-deletion strains and that this phenotype could be partially rescued by ectopic expression of the human DNA-bending protein HMG.

Analyses of DNA looping often rest on the assumption that the proteins mediating the loop are rigid and play no active role in looping other than providing end constraints at DNA binding sites. However, recent experimental [20], [21] and theoretical studies [22] have questioned this assumption and suggest that both protein geometry and flexibility play important roles in the formation of small DNA loops. Protein conformational flexibility can potentially lower the free energy of DNA bending and twisting required for loop formation; if the protein assembly is sufficiently flexible neither enhanced DNA flexibility nor protein-induced bending promoted by additional factors may be needed to stabilize small loops.

A full understanding of the role of DNA looping in gene repression requires a complete and rigorous analysis of the plethora of data obtained from *in-vivo* experiments. Previous analyses [2], [4], [5], [19], [23], [24] have several limitations. In addition to neglecting mechanical contributions from protein flexibility, results are often analyzed by treating DNA looping as being quantitatively equivalent to the related process of DNA cyclization [25]–[31]. We have shown that important distinctions exist between these two processes and that neglecting these differences can potentially lead to misinterpretation of the helical-phase dependence of looping, for example [22]. The major obstacle to quantitatively analyzing experimental data has thus been lack of an accurate and computationally efficient theory for DNA looping [7].

**Figure 1:**
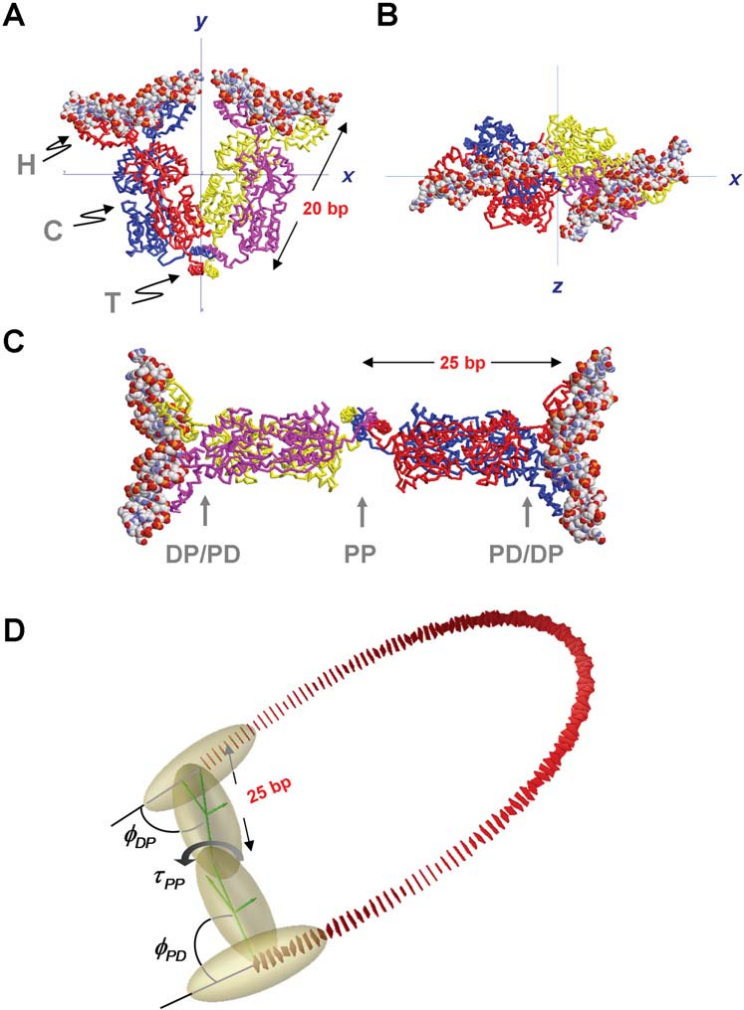
Structures of the *lac*-repressor tetramer complexed with DNA. (A) Crystallographic structure of the “v”-shaped LacR complexed with the symmetric operator sequence GAATTGTGAGCGCTCACAATT (PDF accession number 1LBG) [34], shown along the *z* axis. The α-carbon trace of each lac monomer is rendered in a separate color; DNA segments are shown in a space-filling representation. The three repressor domains are indicated: *H*, headpiece; *C*, core; *T*, tetramerization. The *x* axis is an approximate two-fold or dyad axis in the structure. (B) View of the complex shown in (A) along the *x* axis. Helical axes of the bound DNA segments project slightly (35°) out of the plane of the “v” structure, implying that a small degree of DNA writhe may be induced by LacR-mediated looping. (C) A hypothetical structure of LacR in its extended conformation. This model was generated from the “v”-shaped structure shown in (A) by increasing the interior angle from 60° to ∼180°. The three semi-flexible joints modeling the elastic properties of the tetramer are indicated by vertical arrows. Note that an increase in the length of the LacR major axis from 20 bp to 25 bp occurs when the tetramer isomerizes from the “v-shaped” to the extended structure. (D) Simplified elastic model for LacR and a simulated 137-bp DNA loop mediated by the extended LacR structure. DNA base pairs are represented by rectangular slabs (red). Two sets of coordinate axes (green) represent the local coordinate frames embedded in the protein subunits (gold) that mediate DNA looping. The coupling of protein and DNA geometry is characterized by tilt, roll, and twist values for the DNA-protein, protein-protein, and protein-DNA interfaces. Three of these variables are shown here: the DNA-protein roll angle, *φ_DP_*; the protein-protein twist angle, *τ_PP_*; and the protein-DNA roll angle, *φ_PD_* (see Materials and Methods for details).

Here we describe a comprehensive analysis of the thermodynamics of LacR-mediated repression, including a rigorous statistical-mechanical theory for DNA loop closure [22]. Our treatment considers the mechanics of a protein-mediated loop in terms of a rigid-body approximation that applies both to the base pairs of DNA and to the protein domains that constitute the nucleoprotein assembly. DNA conformations in this model are parameterized using three conventional angular parameters: tilt, roll, and twist, corresponding to rotations of a base pair about the *x, y*, and *z* axes, respectively, of a conventionally chosen local Cartesian-coordinate frame [28]. The geometric arrangement of protein domains is specified by using a similar local coordinate frame fixed within each rigid-body entity of a protein structure (Figure 1). Interaction potentials between base-pair steps and protein domains are taken as quadratic forms in the angular displacements from mechanical equilibrium in the absence of loop-closure constraints. This model therefore allows for conformational flexibility among protein domains and within protein-DNA contacts. We compute the mechanical minimum-energy conformation of the protein-mediated loop and calculate thermodynamic quantities by including thermal fluctuations about this conformation through a harmonic approximation [29]. The approach has many advantages over previous methods in terms of accuracy, computational efficiency, and versatility. It has been applied successfully to the analysis of DNA cyclization data as a special case and provides a basis for understanding the general principles that govern loop-mediated protein-DNA interactions [22]. Swigon et al. [32] recently considered *in-vitro* LacR-mediated DNA looping using a similar strategy [29], although it is not clear to what extent the entropy of particular LacR conformations was considered. Here we extend our approach to investigate LacR-dependent, DNA-loop-regulated gene repression *in vivo*.

Several crystal structures of LacR and the LacR-operator complex, shown in Figure 1, reveal that the repressor can be considered as a dimer of dimers [33], [34]. Each LacR monomer consists of a DNA-binding headpiece, a core domain, and a tetramerization domain. In the crystal structures, a “v”-shaped tetramer is formed from two dimers via a four-helix bundle that comprises the tetramerization domain. This structure has the DNA-binding domains symmetrically placed about a two-fold or dyad rotational axis that lies in the plane of the “v.” The interior angle between the two LacR dimers is about 60° and protein binding induces a local 45° kink in the DNA. However, electron microscopy [35] showed that 44% of LacR in solution is present in an extended conformation (∼180° between the two arms, Figure 1C), with the remaining 56% of complexes in the “v” shape. Additional solution studies support the existence of an extended LacR conformation in small loops containing intrinsically bent DNA sequences [36], [37]. A reasonable way of reconciling the discrepancies is to assume an inherently bistable structure for LacR such that v-shaped and extended conformations can exist in equilibrium. Assuming that the binding affinity to operator DNA is independent of LacR conformation [32], the proportion of each repressor structure in LacR-mediated DNA loops depends on the sum of the free energies arising from DNA and protein distortion during loop formation.

## Results

### Conformational model for the LacR tetramer

Based on the symmetry and modular structure of the LacR tetramer, we model the protein as a dimeric assembly consisting of rigid-body domains connected by semiflexible joints (Figures 1C, D). There are three sets of protein-related rotation angles in addition to those for the DNA dinucleotide steps: two sets for the contacts made by protein domains with the last and first base pairs of the DNA and one set for the contact between protein dimers [22]. These angles describe the kinematics of protein domains joined at the positions shown in Figure 1C. Nearest-neighbor interactions between protein dimers, dinucleotide steps, and between protein domains and DNA are governed by harmonic potentials (see Equation 3 in Materials and Methods) with thermal fluctuations of each DNA base pair expressed in terms of standard deviations of the corresponding angular parameters from their static values. For homogeneous DNA, the standard deviations, σ, for tilt and roll are identical and related to DNA bending persistence length, *P*, by σ = (1/P)^1/2^ where *P* is given in base pairs and *σ* is expressed in radians. The deformability of the protein assembly in this model is similarly specified in terms of standard deviations of the protein-DNA and protein-protein tilt/roll/twist rigid-body parameters.

Following previous observations, we focus on the two canonical LacR geometries: the v-shaped structure characterized by an interior angle of 60° (Figures 1A, B) and the extended tetramer structure, with a 180° interior angle between dimers (Figure 1C). Note, however, that because of strain within the loop, the equilibrium value of this angle is not generally identical to that in the absence of constraints (Figure 1D) [22].

The main thermodynamic quantity to be evaluated is the J factor (see Equation 5 in Materials and Methods), defined by Jacobson and Stockmayer as a measure of the circularization propensity of linear polymer chains [38]. The J factor can be understood in several equivalent ways: (i) as a quantity proportional to the equilibrium constant for formation of a closed chain from an open chain. This process requires association of two chain ends with a consequent reduction from six translational degrees of freedom to three [29]; the J factor thus has units of concentration. With this interpretation it is clear that the free energy of DNA looping is given by ΔG*_loop_* = −k_B_ *T*ln*J*. Note, however, that this formulation of the J factor omits the thermodynamic contribution from protein-DNA association. (ii) The effective concentration of one end of a chain in the vicinity of the other. In the particular case of DNA looping that we discuss here, the J factor is the effective concentration of an auxiliary operator-bound LacR molecule at the primary operator. Due to the tethering effect of DNA looping this concentration can be much higher than the bulk free LacR concentration [2], [7], leading to increased occupancy of the primary site by the repressor and enhanced gene repression. (iii) As the ratio of statistical-mechanical partition functions for closed and open chains [29].

### Multiple DNA loop conformations

**Figure 2:**
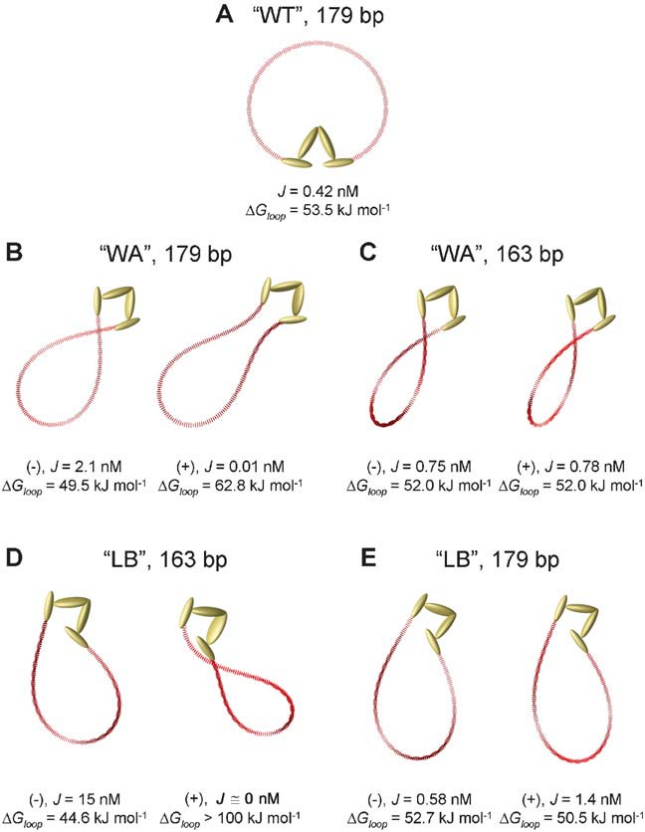
Looped DNA conformations mediated by the v-shaped LacR tetramer structure for 179- and 163-bp DNAs. Three classes of loop conformations: (A) “wrap toward” (“WT”), (B), (C) “wrap away” (“WA”), and (D), (E) “loop beside” (“LB”), are shown along with their respective J factors and Δ*G_loop_* values. At most two alternative loop topoisomers are found for small DNA loops such as those considered here; these correspond to underwound (−) and overwound (+) DNA conformations. The DNA-loop sizes 179 and 163 bp correspond to maxima and minima, respectively, in the DNA-length dependence of *J* for the “WA” or “WT” conformations (see Figure 3A). The 179-bp “WT” conformation shown in (A) is dominated by one planar topoisomer because of its in-phase LacR binding sites at this DNA length; however, there are two alternative 179-bp “WA” conformations, which are shown in (B). The two “WA” topoisomers for 163-bp DNA are essentially isoenergetic and shown in (C); note that the loop crossings differ in topological sign. In contrast, the “LB” conformations (D) and (E) have minimum and maximum *J* values at 179 and 163 bp, respectively. Only the 179-bp (−) “LB” topoisomer shown in (D) is populated to any appreciable extent at thermal equilibrium, whereas both (−) and (+) forms have similar free energies in the case of 163-bp “LB” loops.

For the v-shaped LacR, there are three classes of mechanical-equilibrium looped conformations (Figure 2) [33], denoted “WT” (“wrapping towards”), “WA” (“wrapping away”), and “LB” (“looping beside”), depending on the DNA trajectory's approach relative to the inside or outside of the “v.” Because a pseudo-dyad axis is shared by the LacR DNA-binding headpiece and the operator sequence, operator-binding affinity should be independent of the local orientation of the DNA binding site. This property is expected even though the operator sequence may not itself be palindromic. In general, each class consists of a pair of overtwisted and undertwisted topoisomer solutions [22]. These three classes of loop conformations were also found by Olson and coworkers [32], described using different nomenclature [39].

For our calculations, we used a planar v-shaped structure to represent the repressor. In the crystal structure of the LacR-operator complex, the helical axes of the operator sites do not lie in the mean plane of the repressor structure and are instead displaced by about 20 degrees (Figure 1B). However, we found that J factors were relatively insensitive to this angle (see Figure S1). In contrast to the v-shaped LacR structure, there is only one class of “simple loops” (“SL”) formed by the extended LacR tetramer (Figure 1D) [22], [32].

**Figure 3:**
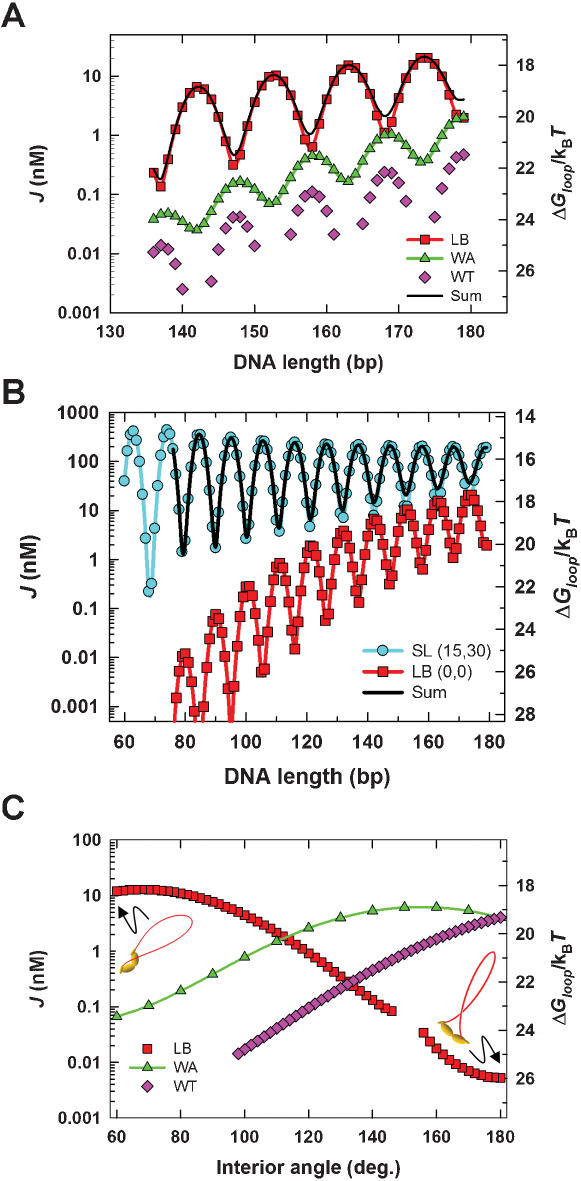
J factor and Δ*G_loop_* values versus DNA length or LacR interior angle for four classes of loop conformations. (A) Comparison of J factors and Δ*G_loop_* for the three classes of loop conformations mediated by the v-shaped *lac* repressor. Protein assemblies are taken to be rigid (i.e., DNA-protein, protein-protein, and protein-DNA flexibility parameters were all set to 0). (B) Length dependence of J factors and Δ*G_loop_* for the extended (SL) and LB conformations. Protein-flexibility parameters are given in parentheses as (*σ_θ_^PP^ = σ_φ_^PP^ = σ_τ_^PP^*, *σ_θ_^DP^ = σ_θ_^PD^ = σ_φ_^DP^ = σ_φ_^PD^ = σ_τ_^PD^ = σ_τ_^DP^*). Taken together with (A), the J-factor length dependence shows that the extended LacR conformation dominates all of the v-shaped forms for loops smaller than 180 bp. (C) The dependence of *J* and Δ*G_loop_* values on the interior angle between LacR domains is shown for three classes of 153-bp loops as the repressor structure opens from the v-shape (60°) to an extended form (180°). Protein assemblies were taken to be rigid, as in (A). WA and WT loops become degenerate at large angles, which can be seen from the identical J factors attained with the extended form of LacR. A small difference (∼1.5k_B_ *T*) between the asymptotic Δ*G_loop_* value for WA and WT conformations in (C) and the corresponding value on the SL curve in (B) is due to differences in protein flexibility and tetramer dimensions (dimer major-axis length of 25 bp in (B) versus 20 bp in (C)). Because of broken symmetry, LB loops adopt a highly strained conformation as the interior angle approaches 180°. For comparison, projections of 3-d conformations for LB loops with interior-angle values of 60° and 180° are shown as insets. Gaps in the curves indicate that no stable mechanical-equilibrium conformations were found for “LB” loops when the interior angle was between 146° and 156°, nor for “WT” loops having interior-angle values less than 98°. This behavior is characteristic of abrupt transitions between mechanical minima, usually when a loop bifurcates to either an over-twisted or under-twisted conformation. Without a stable mechanical-equilibrium conformation, the perturbation method employed in our statistical-mechanical theory cannot be applied.

### Helical dependence of DNA looping for different LacR conformations

The computed J factors for the three classes of v-shaped loop conformations are shown as functions of loop size (or DNA length) in Figure 3A. Values of *J* for particular conformations and corresponding values of the looping free energy are also given in Figure 2. Remarkably, the LB conformation has the largest *J* value among the three classes of v-shaped protein structures and dominates the distribution (Figure 3A). There is a one-half-turn difference in the helical-phase dependence of *J* for LB conformations relative to those for the WA and WT conformations. The difference in phasing arises because the LB conformation involves a 180-degree rotation of one operator element about the sequence dyad with respect to the WA and WT conformations. Moreover, the amplitude of the helical-phase dependence for the LB conformation is significantly larger than that for the WA conformation. This dependence of J-factor amplitude on loop conformation militates against the general use of empirical formulas based on DNA-cyclization theory to estimate the DNA torsional rigidity [19], [24], [28] and underscores the need to explicitly consider protein geometry and mechanics in models of DNA looping [22].

**Figure 4:**
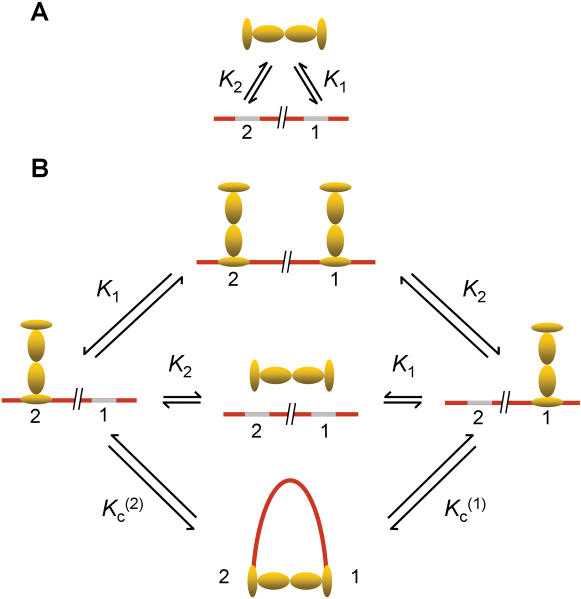
Chemical equilibria among LacR-operator association states assuming a single looped conformation for the LacR-DNA complex. (A) A LacR tetramer can bind to two cognate sites with different affinities, for which *K_1_* and *K_2_* are apparent dissociation constants. Assuming site “1” is the primary site near the promoter of the *lac* operon, its occupancy by LacR prevents RNA polymerase from binding to the promoter, blocking transcription of genes under its control. (B) Coupled equilbria involving different LacR-DNA complexes. Here *K_c_^(1)^* and *K_c_^(2)^* denote the unimolecular association constants associated with DNA looping and are related to *K_1_, K_2_*, λ, and *J* by Equation 9 (see Materials and Methods).

The length dependence of *J* for SL loops mediated by the extended LacR structure is shown in Figure 3B. J factors for the SL conformation greatly exceed those for even the most thermodynamically favorable v-shaped conformation, LB. This difference between SL and LB loops is particularly pronounced for DNA loop sizes less than 100 bp, which is the range used in many studies of *in-vivo* gene repression regulated by DNA looping. The comparison in Figure 3B involves different protein-flexibility parameters for the two tetramer structures. Because the v-shaped tetramer is locked in place by interactions between the central domains of the two LacR dimers, it is likely that conformational flexibility in this compact conformation is substantially less than that of the extended conformation in which these interactions have been broken [34], [40], [41].

Figure 3B also shows that differences in protein structure and conformational flexibility dramatically alter the balance between elastic energy and chain entropy in loop formation as a function of DNA length [22], [29]. There is a small, but significant, decrease in chain entropy with increasing loop size for the formation of SL-class loops, indicated by the decay of J-factor peaks with increasing DNA length. This increase in looping free energy stands in contrast to other results [32]. In the case of loops mediated by the v-shaped LacR structure, however, J factors increase with DNA length, demonstrating that these structures are determined by loop elastic energy. The phase dependence of the SL conformation is the same as that for the WT and WA structures and is one-half-turn out of phase relative to the LB conformation. This ∼5-bp difference in phasing between SL and LB loops implies that loop sizes that are J-factor minima in the SL length dependence closely coincide with J-factor maxima for the LB conformation.

Solutions for WT and WA conformations, but not those for LB, are expected to approach those for the extended repressor conformation in the limit where the LacR interior angle approaches 180 degrees. We examined J factors for a 153-bp loop formed by each of the three v-shaped structures as a function of the interior angle (Figure 3C). The results show that as the angle opens up from the near-crystallographic value of 60 degrees to the fully extended state (180 degrees, see Figure 1C), the LB conformation becomes increasingly unfavorable whereas WA and WT structures become increasingly favorable. Fully extended, the WA and WT structures are degenerate; as expected, they have identical *J* values and looping free energies. Unlike the WA and WT loops, increasing the interior angle drives the “LB” structure toward the conformation of a loop with approximately parallel ends (in contrast to the approximately antiparallel ends in Figure 1D). Such strained conformations have dramatically diminished J factors.

The J factor is a direct measure of the relative proportions of particular looped conformations at thermodynamic equilibrium. In principle, J factors for all classes of loop conformations should be taken into account in calculating the free energy of LacR-mediated loop formation. However, based on the comparative magnitude of J factors for the SL and v-shaped repressor structures (Figure 3B), we chose to simplify our analysis of *in-vivo* repression data by using *J* values for the SL loop class exclusively. In doing so we neglected the possible free-energy difference for LacR tetramers in the two conformations, which has not been accurately measured, but may be relatively small [32], [35].

### Thermodynamic model for *in-vivo* gene repression regulated by LacR-mediated DNA looping

The energetics of loop formation depend not only on the geometry and mechanical properties of protein and DNA expressed in terms of the J factor, but also on the binding equilibria relating different protein-DNA association states (Figure 4). In experiments of Müller *et al*. [5], the observable quantity is expression of a reporter gene (e.g., β-galactosidase) as a function of variables such as operator spacing or operator affinity for LacR. To quantitatively analyze gene repression based on a model for DNA looping, we assume that the rate of reporter-gene expression is under thermodynamic control, namely, proportional to the probability that the primary operator is unbound. This assumption has been used in previous analyses of LacR-mediated gene repression [4], [42], [43].

**Figure 5:**
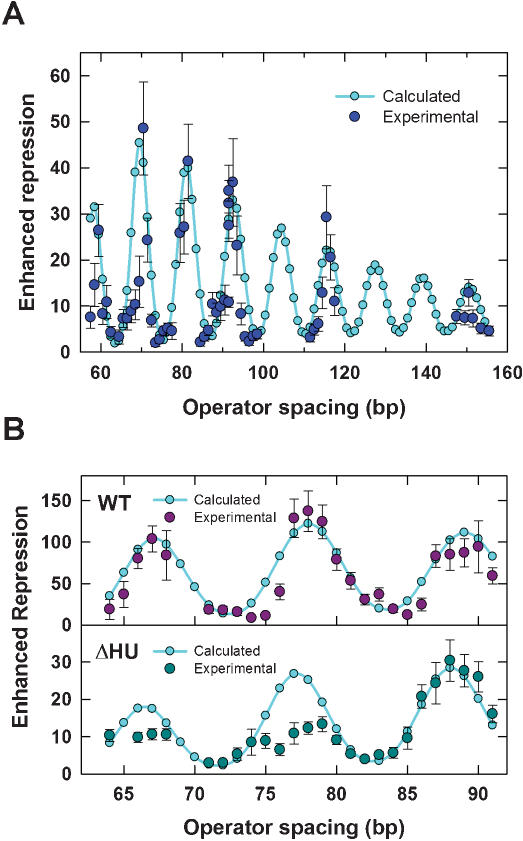
Best fit of the thermodynamic model to *in-vivo* repression data. Best-fit values of the adjustable parameters for all data sets are given in Table 1. (A) *In-vivo* repression data from Figure 3A of Müller et al. [5] and optimized fit to Equation 1. (B) *In-vivo* repression data of Becker et al. [19] for wild-type (“WT”) and HU-deletion (ΔHU) *E. coli* strains along with their respective optimized fits.

Based on the equilibria shown in Figure 4 and derivations given in Materials and Methods, the enhancement of gene repression by DNA looping, *R*, is calculated according to the formula
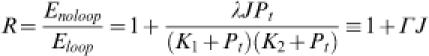
1where *E_noloop_* and *E_loop_* denote rates of gene expression in the absence of DNA looping (i.e., deletion of the auxiliary site indicated by site 2 in Figure 4), and that in its presence, respectively. In Equation 1 *P_t_* is the LacR-tetramer concentration in the cell and *K_1_* and *K_2_* are equilibrium dissociation constants of LacR for the primary and auxiliary operator sites, respectively. The dimensionless parameter λ mainly accounts for possible allosteric effects when one LacR tetramer associates with two DNA sites, with λ>1 for cooperative binding and λ<1 for anti-cooperative binding (see Equation 9 in Materials and Methods). We chose λ = 1 throughout because formation of the bidentate LacR-operator complex is non-cooperative [44]. The factor Γ contains all information concerning protein-DNA association exclusive of the looping contribution. In the special case of strong operator sites, i.e., *K_1_, K_2_*<<*P_t_*, the enhanced repression in Equation 1 can be simplified to R = 1+*J/P_t_*. This expression confirms the notion that the role of DNA looping is to increase the local protein concentration, thereby enhancing gene repression. It also shows that the enhancement increases with decreasing protein concentration, a conclusion discussed in greater detail below.

In some experiments [5], repression was determined from the ratio of β-galactosidase activity measured for *E. coli* strains lacking a plasmid-borne LacR expression system to that for strains carrying the expression plasmid. Throughout our data analysis we adopt the definition of enhanced repression given in Equation (1), which is more appropriate for characterizing effects of DNA looping. This enhanced repression is the ratio of measured reporter activities for a construct in which the auxiliary operator has been deleted to that for a construct containing both primary and auxiliary operators. Therefore, to calculate *R*, the repression values of Müller *et al*. [5] were normalized relative to the measured value for a primary operator-only construct (120, see Figure 2 in [5]) under identical conditions. The resulting *R* values were then subjected to a multi-parameter curve-fitting analysis described below.

### Analysis of *in-vivo* LacR-mediated gene repression based on DNA looping

Müller *et al*. [5] demonstrated a dramatic dependence of repression on helical phasing in systematic measurements of LacR-dependent gene repression at incremental operator spacings between 57.5 bp and 155.5 bp (the non-integral value is due to a 1-bp length difference between operator sequences). To fit these data using a non-linear least-squares method, we chose four adjustable parameters in our model: DNA helical repeat, DNA persistence length (or bending flexibility), DNA torsional rigidity (or twisting flexibility), and protein flexibility. All four parameters implicitly determine the value of the J factor in Equation 1. Protein-flexibility parameters (standard deviation values corresponding to angle fluctuations) for both protein-DNA and protein-protein contacts (Figure 1) assumed identical values and Γ (Table 1) was computed using reported values of *P_t_, K_1_* and *K_2_* [45], [46] according to Equation 1. Note that a single value of the factor Γ applies to all values of the operator spacing in this model. Optimization over the four adjustable parameters was carried out using a simplex algorithm minimizing the following target function
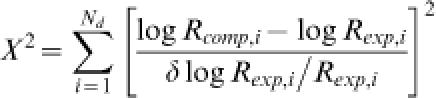
2where *R_comp,i_, R_exp,i_* denote the computed and experimental enhanced-repression values, respectively. To avoid overfitting to low experimental repression values (Figure 5), we chose the weight *δ*log*R_exp,i_/R_exp,i_* = *δi*/(*R_exp,i_*)^2^ in the least-squares fit, with *δ_i_* the reported experimental error for the *i*-th data point. The total number of experimental data points, *N_d_*, was equal to 51. We obtained the fit to the experimental data shown in Figure 5A with the corresponding best-fit adjustable parameters given in Table 1; experimental and fitted enhanced-repression values as well as computed J factors for all of the analyses described can be found in Tables S1 and S2.

**Figure 6:**
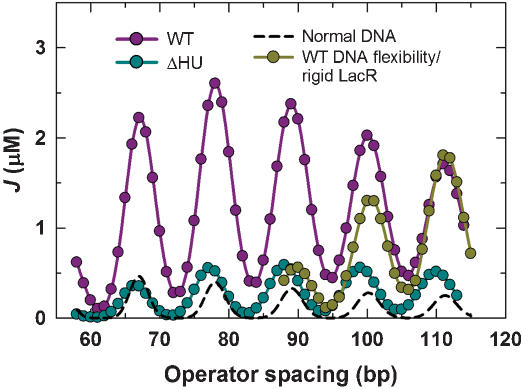
J factor versus operator spacing under different intracellular conditions. The *J* values corresponding to wild-type, WT, or HU-deletion, ΔHU, *E. coli* strains were calculated using the respective best-fit parameters given in Table 1. J factors corresponding to LacR-mediated loops having normal DNA elasticity were computed using canonical parameters for DNA persistence length (150 bp) and torsional rigidity (2.4 10^−19^ erg cm), and a LacR flexibility parameter identical to that for the wild-type strain (19°). Calculations of the J factor for the case of a rigid extended LacR conformation used the same parameters as for the wild-type strain except for a protein-flexibility parameter set equal to 2.0°.

**Table 1:**
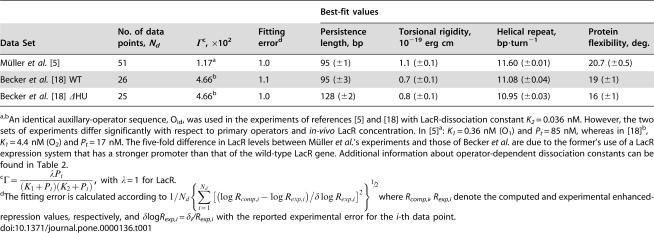
Best-fit Values of Adjustable Parameters for LacR-loop-mediated Repression Data.

				Best-fit values			
Data Set	No. of data points,* N_d_*	Γ^c^, ×10^2^	Fitting error^d^	Persistence length, bp	Torsional rigidity, 10^−19^ erg cm	Helical repeat, bp·turn^−1^	Protein flexibility, deg.
Müller *et al.* [5]	51	1.17^a^	1.0	95 (±1)	1.1 (±0.1)	11.60 (±0.01)	20.7 (±0.5)
Becker *et al.* [18] WT	26	4.66^b^	1.1	95 (±3)	0.7 (±0.1)	11.08 (±0.04)	19 (±1)
Becker *et al.* [18] ΔHU	25	4.66^b^	1.0	128 (±2)	0.8 (±0.1)	10.95 (±0.03)	16 (±1)

a,b An identical auxillary-operator sequence, O_id_, was used in the experiments of references [5] and [18] with LacR-dissociation constant *K_2_* = 0.036 nM. However, the two sets of experiments differ significantly with respect to primary operators and *in-vivo* LacR concentration. In [5] ^a^: *K_1_* = 0.36 nM (O_1_) and *P_t_* = 85 nM, whereas in [18] ^b^, *K_1_* = 4.4 nM (O_2_) and *P_t_* = 17 nM. The five-fold difference in LacR levels between Müller *et al.*'s experiments and those of Becker *et al.* are due to the former's use of a LacR expression system that has a stronger promoter than that of the wild-type LacR gene. Additional information about operator-dependent dissociation constants can be found in Table 2.

c
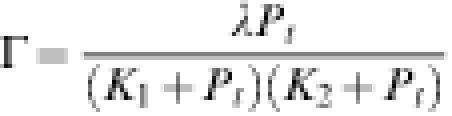
, with λ = 1 for LacR.

d The fitting error is calculated according to 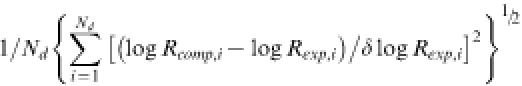
where *R_comp,i_, R_exp,i_* denote the computed and experimental enhanced-repression values, respectively, and δlog*R_exp,i_ = *δ*_i_/R_exp,i_* with the reported experimental error for the *i*-th data point.

As shown in Table 1, values of the persistence length and torsional rigidity are respectively reduced by about 37% and more than 50% relative to their corresponding canonical values *in vitro* [47], [48]. The fitted value of the DNA helical repeat, 11.60 (±0.01) bp/turn, is consistent with previously reported *in-vivo* values [6] and is larger than that for topologically unconstrained DNA free in solution (≈10.5 bp turn^−1^) because of DNA unwinding that accompanies negative supercoiling *in vivo* [49]. Our quantitative analysis of these gene-repression data first shows that the high degree of protein flexibility (20.7±0.5°) cannot completely compensate the requirement for increased DNA flexibility *in vivo*. The high overall flexibility of the nucleoprotein assembly is reflected in the decay of repression peaks with operator spacings above 70 bp. This entropy-dominated effect in DNA looping is a unique feature of statistical-mechanical models [22], which take full account of DNA and protein flexibilities.

The strong agreement between calculated repression values and experimental data quantitatively verifies the role of DNA looping in *lac* gene regulation. In particular, our model explains the optimal O_1_–O_3_ separation of 92 bp observed in the wild-type operon, which coincides with a strong peak in the enhanced- repression curve (Figure 5A). Once all of the adjustable parameters in the model were determined from the fitting procedure, we were able to compare predicted repression values with additional experimental measurements. Müller et al. also investigated the effects of operator quality at fixed operator spacing (∼92 bp) [5]. In this experiment, the high-affinity auxiliary operator (O_id_) used in the previous analysis was replaced by three different operator sites with weaker affinities for LacR. Table 2 shows that measured repression values and those calculated according to Equation 1 also give very good agreement.

**Table 2:**
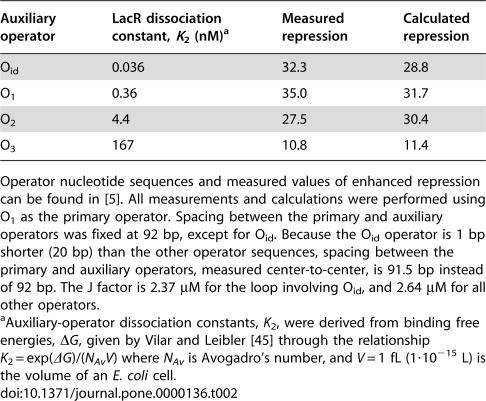
Effect of operator quality on DNA looping and gene repression at approximately constant helical phasing.

Auxiliary operator	LacR dissociation constant, *K*_2_ (nM)^a^	Measured repression	Calculated repression
O_id_	0.036	32.3	28.8
O_1_	0.36	35.0	31.7
O_2_	4.4	27.5	30.4
O_3_	167	10.8	11.4

Operator nucleotide sequences and measured values of enhanced repression can be found in [5]. All measurements and calculations were performed using O_1_ as the primary operator. Spacing between the primary and auxiliary operators was fixed at 92 bp, except for O_id_. Because the O_id_ operator is 1 bp shorter (20 bp) than the other operator sequences, spacing between the primary and auxiliary operators, measured center-to-center, is 91.5 bp instead of 92 bp. The J factor is 2.37 µM for the loop involving O_id_, and 2.64 µM for all other operators.

a Auxiliary-operator dissociation constants, *K*_2_, were derived from binding free energies, *ΔG*, given by Vilar and Leibler [45] through the relationship *K*_2_ = exp(Δ*G*)/(*N_Av_V*) where *N_Av_* is Avogadro's number, and *V* = 1 fL (1·10^−15^ L) is the volume of an *E. coli* cell.

Recent studies suggest that additional factors responsible for enhanced DNA flexibility, such as HU protein, may play an important role in facilitating loop-mediated gene regulation [19]. We investigated the role of HU in modulating DNA looping by analyzing the comparative LacR-dependent repression data of Becker *et al.* for *E. coli* strains expressing wild-type levels of HU protein and a mutant lacking HU [19]. Our analysis of these data sets used a value of Γ that was adjusted to reflect differences in operator affinities and LacR concentration relative to the Müller *et al.* experiments (Table 1). The resulting fit to the wild-type data in Figure 5B (upper panel) shows excellent agreement between the theory and experimental data and gives a low value for the persistence length, identical to that obtained in the fit to the Müller *et al.* data (Table 1). Compared with the Müller *et al.* results, Becker *et al.*'s enhanced-repression values are significantly greater (about three-fold as judged by the repression peaks), whereas the amplitude of the helical dependence is about two-fold smaller. The larger value of Γ in Becker *et al.*'s experiments, due to five-fold lower LacR expression (see Table 1, also Equation 1), is mainly responsible for the increased enhancement, whereas the diminished amplitude results from lower DNA torsional rigidity, which is decreased even further relative to its *in-vitro* value compared with the Müller *et al*. results. The DNA helical-repeat value (11.1 bp turn^−1^) is consistent with lower levels of negative supercoiling than that in the earlier study [50]. Differences in helical-repeat values between the two data sets are not surprising, given the complex dependence of supercoiling on cellular physiology [51], [52] and differences in the *E. coli* strains and DNA constructs used [5], [19]. Specifically, in Müller *et al.*'s experiments DNA-looping assays involved operator sequences that were located on the *E. coli* chromosome, whereas in Becker *et al.*'s experiments, operator sequences resided on single-copy F′ episomes. The DNA sequences between the operator sites were substantially similar in the two studies; none of the intervening DNA sequences contained any known intrinsic bends or other unusual features.

Using the same value of Γ estimated for the wild-type case, we then fitted Becker *et al.*'s data for repression in an HU deletion strain (Figure 5B, bottom panel). There was a marked increase in the best-fit DNA bending rigidity (Table 1), bringing this value into a range compatible with DNA molecules at moderate ionic strength *in vitro* [47], [48]. Although the ΔHU persistence-length value (128±2 bp) is somewhat smaller than that normally given for mixed-sequence DNA in solution, it is equal within experimental uncertainty to values measured by rotational diffusion experiments at high salt (129 bp in 110 mM Na^+^/10 mM Mg^2+^) [53]. The abundance of multivalent cations and polyamines *in vivo* is expected to have significant effects on DNA elasticity [54]; however, it is also possible that non-specific binding of other architectural DNA-bending proteins present in the cell or sequence-dependent variations in bending flexibility in the region between operator sites may contribute to the slightly reduced persistence length.

Our torsional-rigidity values in the presence and absence of HU compare favorably with those estimated by Becker *et al.* using an empirical formula that contained torsional elasticity only. The model described here takes LacR structure and both bending and torsional flexibility of the entire nucleoprotein assembly into account and thus provides rigorous and quantitative evidence for a direct functional role of HU protein on DNA elasticity and loop-dependent interactions *in vivo*.

### Biological consequences of DNA looping

**Figure 7:**
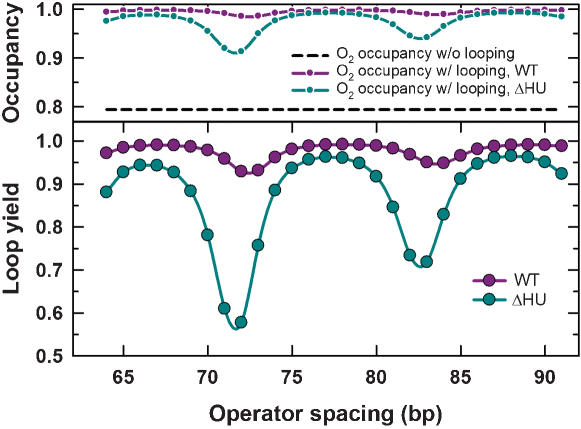
Predicted repressor occupancy of the primary operator and loop yield as a function of operator spacing corresponding to the data of Becker *et al.* [**19**]. Upper panel: the proportion of the primary operator site (O_2_) bound by LacR for wild-type and HU-deletion strains computed from their respective best-fit parameters using formula 1−*E_loop_* (see Equation 13). The occupancy of the primary operator in the absence of the looping contribution is shown for reference (dashed line). Lower panel: the loop yield computed using Equation 10 for both wild-type and HU-deletion strains. *J* values used in these calculations are those given in Figure 6.

One of the most frequently cited biological roles for DNA looping is to raise the local concentration of a regulatory protein in the vicinity of a promoter element [2], [7]. Our rigorous analysis confirms this picture for LacR-mediated looping. As shown in Figure 6, DNA looping in HU-containing wild-type cells boosts the LacR concentration (J factor) at the primary operator (O_1_) from its bulk value of 0.017 µM to between 0.28 and 2.6 µM. This effect raises the occupancy of the primary operator, the fraction of primary operator sequences bound by LacR, from 0.79 to between 0.985 and 0.998, a value essentially insensitive to helical phasing (Figure 7, upper panel). Such pronounced enhancement of operator occupancy has the consequence of decreasing the expression rate of β-galactosidase (molecules per hour per cell) [5], [55] from 1,300 to a range of 12 to 90. In wild-type *E. coli* strains with an O_1_–O_3_ operator pair separated by 92 bp (Table 2), the predicted O_1_ occupancy is 0.9986 (equivalent to about 8 β-galactosidase molecules per hour per cell), in excellent agreement with direct *in-vivo* measurements [3], [56].

For a two-operator system, occupancy of the primary operator (Figure 7, upper panel) involves a looped state and two unlooped states (Figure 4). To relate the enhanced operator occupancy and gene repression to DNA looping, we calculated the loop yield (Figure 7, bottom panel), which is the proportion of looped states relative to all possible states. The loop yield directly correlates with the J factor, operator occupancy, and enhanced gene repression as demonstrated by their identical dependence on DNA helical phase (Figures 5B, 6, and 7). Furthermore, the high loop yield (0.929–0.992) confirms that enhanced gene repression is almost exclusively attributable to DNA looping.

**Figure:**
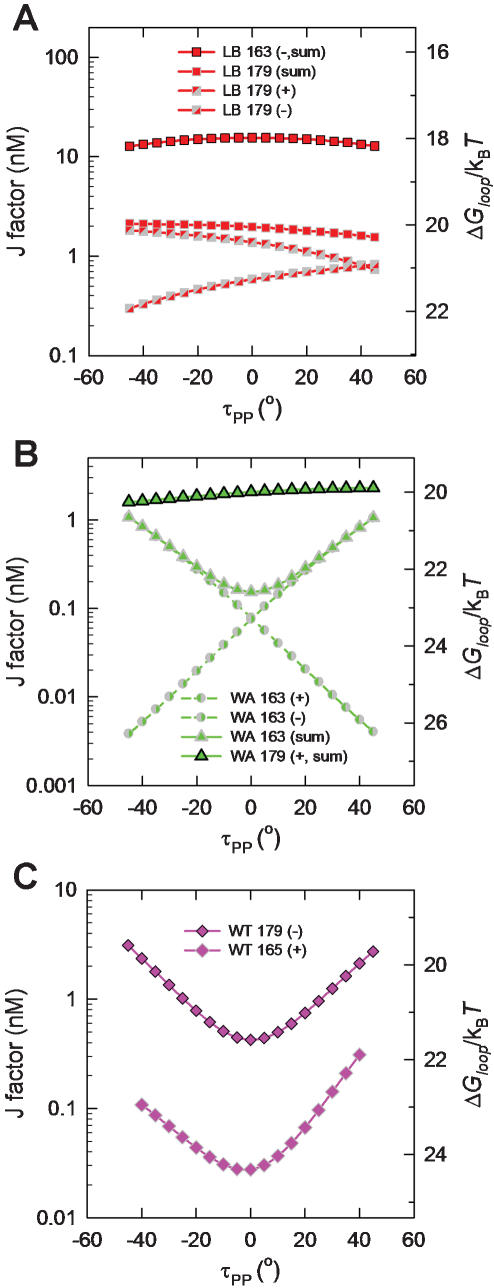


In the absence of HU, increases in effective DNA bending and protein rigidities reduce measured gene repression by up to twelve-fold, depending on operator spacing, with an average reduction of 5.6-fold (Figure 5B). The effect of HU is also apparent from decreases in J factor (Figure 6), operator occupancy, and loop yield (Figure 7) for the HU-deficient *E. coli* strain compared with wild-type. To put this finding in perspective, we calculated J-factor values using a canonical DNA persistence length of 150 bp (Figure 6). As expected, *J* values are much smaller than those obtained *in vivo* in the presence of HU [5], [19]. These comparisons quantitatively confirm HU's putative role in facilitating the formation of small DNA loops *in vivo*. DNA torsional rigidity *in vivo* is substantially reduced relative to the *in-vitro* value and is not significantly affected by the presence of HU protein (Table 1). The basis of HU's differential effect on bending and torsional rigidities is not clear. We speculate that DNA supercoiling *in vivo* may enhance nonlinearities in DNA torsional elasticity [11], [57] and that this effect is largely independent of HU expression.

Dynamic DNA bending induced by HU protein is not the only factor that reduces the thermodynamic cost of small-loop formation, however. Significant helical-phase-dependent enhanced repression remains even when HU is deleted (Figure 5B). Consistent with this observation, predicted values of operator occupancy (Figure 7, upper panel) are significantly greater than that in the absence of DNA looping and the major proportion of DNA is expected to be in the looped state (Figure 7, bottom panel). While investigating other contributions to small-loop formation, we noticed that the effective conformational flexibility of LacR is only marginally reduced (from 19° to 16°) in the absence of HU (Table 1). This slight change in the protein-flexibility parameter is probably caused by differing extents of LacR deformation in forming DNA loops and accompanying nonlinearity of protein elasticity.

We assessed the specific contribution of protein flexibility to loop formation by comparing the J factors calculated for a flexible protein assembly with those of a rigid extended LacR structure (Figure 6), assuming the same DNA rigidities as those in the HU-containing wild-type strain. Interestingly, we could obtain solutions only in cases where the operator spacing was greater than 88 bp, indicating that DNA loops smaller than this size have highly unfavorable looping free energies. In the range from 88 to 100 bp, J factors with rigid LacR tetramers are lower than those with flexible tetramer assemblies. This comparison demonstrates the crucial role of protein flexibility in forming small DNA loops. In contrast, above ∼108 bp, high protein flexibility makes DNA looping unfavorable due to increased entropy loss [22]. Taken together, we conclude from these results that protein structure, protein conformational flexibility, and DNA flexibility induced by non-specific protein-DNA interactions such as those with HU, all contribute significantly to the formation of small DNA loops widely observed *in vivo*.

## Discussion

Gene repression in the *lac* system has become a textbook example of how DNA looping modulates the local concentration of a regulatory protein in the vicinity of a promoter. Intensive study over the last three decades has led to a wealth of information about the thermodynamics of *lac* repressor's interaction with single wild-type and mutant operator sequences and the dependence of gene repression on operator spacing. However, the quantitative effect of DNA looping on LacR-mediated gene regulation has been highly controversial. Here we provide a novel analysis of *in-vivo* gene repression from first principles based on a rigorous statistical-mechanical model of DNA looping.

We analyzed two independent data sets that characterize the dependence of LacR-mediated repression on inter-operator spacing: those of Müller *et al.* [5], and the data of Becker *et al.* [19]. Both sets of experiments systematically cover overlapping ranges of operator-site spacing that span at least two full helical turns. Moreover, both studies were carried out using constructs in which the CAP-binding site located near the promoter was abolished, eliminating the need to take possible CAP-dependent DNA bending or CAP-LacR interactions into account in the calculation of loop free energy [58], [59]. We chose on this basis not to analyze the classic results of Law *et al.* [4] because those experiments were done with regulatory regions that included an intact CAP-binding site.

The excellent agreement between experiments and this analysis validates the dominance of the extended LacR structure in DNA looping *in vivo* over the v-shaped LacR structure widely observed *in vitro*. This conclusion is largely consistent with analyses of both the Müller *et al.* and Becker *et al.* results by Saiz et al. [24], [60], in which a single LacR conformation was found to populate ∼80% of the DNA loops formed. The remaining proportion was proposed to be in an alternative conformation, although the details of these two distinct conformations could not be determined from their analysis. The small discrepancy with the present work regarding the existence of this alternative conformation *in vivo* may be due to the simplified semi-quantitative model used by Saiz et al., in which DNA bending flexibility and LacR structure and conformational flexibility were not included. Although a small contribution to looping from the “v-shaped” LacR conformation cannot be ruled out, the extended conformation alone is sufficient to account for all of the *in-vivo* repression data. In the case of the Müller *et al.* data set, our computed repression peaks are somewhat broader than the corresponding experimental ones (see Figure 5A). For unknown reasons, the difference in theoretical and experimental peak widths is a particular feature of the Müller *et al.* data set; there is no obvious broadening of the computed repression peaks relative to those in the Becker *et al.* data (Figure 5B).

Several studies have investigated the specific role of DNA architectural proteins such as HU and HMG in enhancing apparent DNA bending and twisting flexibilities *in vitro* and *in vivo* [14]–[17], [19], [61], [62]. HU is an abundant protein in *E. coli*, present at levels of 60,000 copies per wild-type cell [63], or equivalently, about one HU dimer per 100 bp DNA. *In vitro*, DNA fragments as short as 100 bp can be readily circularized by DNA ligase in the presence of HU [15], albeit at HU:DNA ratios that significantly exceed the *in-vivo* value. Nevertheless, abolishing HU protein in a deletion strain dramatically reduces gene repression mediated by LacR (Figure 5B) and directly correlates with decreased apparent DNA bending flexibility. Our results therefore suggest that HU plays an important role in regulating DNA bendability *in vivo*.

Because DNA looping is an essential mechanistic feature of many biological processes including transcription, DNA replication, recombination, and repair [64], the principles that govern DNA looping in the *lac* system generally apply to a very large class of problems in biological regulation and function. The quantitative approach described here should open the way to rigorous *in-vitro* and *in-vivo* characterization of this biologically important class of regulatory mechanisms. An even more comprehensive picture of DNA looping will emerge once a more complete understanding of the kinetics of loop formation is attained [20], [55], [65].

## Materials and Methods

### Statistical-Mechanical Theory of DNA Looping and Computational Methods

Details of the theory have been published elsewhere [22], [29]; thus, only a summary of salient features is presented here. We simplify the structure of a protein-mediated DNA loop by treating the nucleoprotein assembly as a connected chain of rigid bodies. The Hamiltonian for a free chain in the absence of constraints is
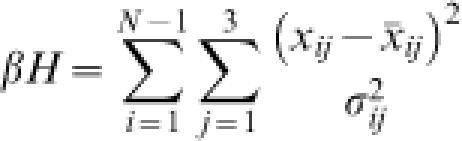
3where *X_ij_*(*j* = 1,…,3) denotes the instantaneous rotation angle (tilt, roll, or twist) of the *i*-th rigid body relative to the (*i*-1)-st one in the presence of thermal fluctuations characterized by *σ_ij_*, and *x_ij_* is the corresponding equilibrium angle. Here *N* is the total number of rigid bodies in the chain and *β* = 1/k_B_ *T*. The Hamiltonian for a closed loop is also described by Equation (3), but subject to six constraints due to chain closure [29], i.e.,
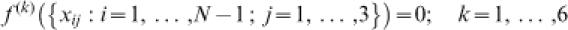
4which are nonlinear functions of the angular parameters. After finding the mechanical equilibrium conformation of the closed loop with minimum elastic energy, the J factor is calculated using the formula [29]

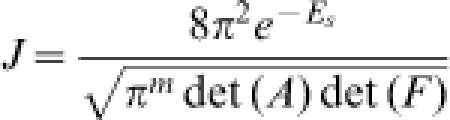
5where *E_s_* is the mechanical elastic energy of the loop and *m* = 6. Here ***A*** and ***F*** are matrices with dimensions (3*N*−3)×(3*N*−3) and 6×6, respectively, whose elements are functions of the thermal fluctuations *σ_ij_* and the first and second derivatives of the constraint functions (left side of Equation 4) with respect to angular parameters at the mechanical-equilibrium conformation.

Unless noted otherwise, all calculations used canonical parameters for duplex DNA: helical twist τ_0_ = 34.45°, a sequence-independent twist-angle standard deviation, or twisting flexibility, σ_τ_ = 4.388°, and standard deviations, or bending flexibilities, for all tilt (*θ*)and roll (*φ*) angles, σ_θ_ and σ_φ_, respectively, of 4.678° (equivalent to an isotropically flexible DNA chain with a persistence length of 150 bp). Average values of tilt and roll for DNA were taken to be zero. To model the DNA loops mediated by the v-shaped protein conformation, we used the following angular parameters for the mechanical-equilibrium conformations of the LacR tetramer [22]: *φ_DP_* = *φ_PD_* = 67.5°, *φ_PP_* = 120° for “WA;” *φ_DP_* = *φ_PD_* = 67.5°, *φ_PP_* = −120° for “WT;” and *φ_DP_* = −67.5°, *φ_PD_* = 67.5°, *φ_PP_* = 120°, *τ_DP_* = 180°+34.45° = 214.45° for “LB” [34], [35]. For the extended LacR conformation, *φ_DP_* = *φ_PD_* = 67.5°, *φ_PP_* = *τ_PP_* = 0 [35]. The subscripts specify angular-parameter values for contacts between the protein and the last (DP) and first (PD) base pairs of the DNA loop or between the two protein domains (PP). Note that these parameters take protein-induced DNA bending (≈45°) at the operator sites into account. Slightly different values for the length of the major LacR-dimer axis were used for the v-shaped and extended LacR conformations: 20 bp and 25 bp, respectively [35]. J-factor computations were carried out on a 2.8-GHz Pentium-4 CPU with 2 GBytes RAM. Geometries of optimized LacR-DNA looped conformations were visualized using the OpenDX data visualization package (http://www.opendx.org/). Fortran-90 and C-language source code is available upon request.

### Analysis of *in-vivo* LacR-mediated repression

We assume that protein-protein association is maintained under all conditions and that all specific protein-DNA interactions are accounted for in the equilibria shown in Figure 4. Intermolecular DNA associations have also been observed in some *in-vitro* experiments [36], [66] because of high DNA concentrations that were used. *In vivo*, these bimolecular associations are unlikely and are therefore not shown in Figure 4.

Take *p*_0_ to be the concentration of free LacR tetramer, and let *d*_0_, *d*_1_, *d*_2_, *d*_12_, and *d_c_* designate the concentrations of free DNA, DNA with site “1” bound, DNA with site “2” bound, doubly-bound (unlooped) DNA, and the closed loop, respectively. Then the following equations hold
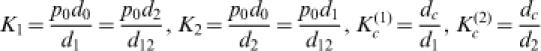
6with
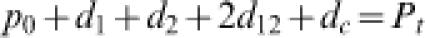
7and
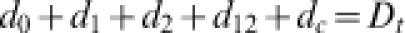
8where *D_t_* and *P_t_* are the total DNA and protein concentrations, respectively. Note that *K*_1_ and *K*_2_ are dissociation constants, whereas the loop-closure constants *K*_c_ describe an association reaction. By analogy with its definition in DNA cyclization [25], [27], the J factor is the ratio of unimolecular association constant to that of bimolecular association, i.e.,
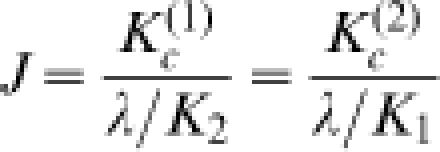
9


Here we include λ as a dimensionless factor that accounts for possible changes in affinity that could accompany three effects: (1) allosteric binding when one LacR tetramer associates with two DNA sites, (2) nonspecific DNA binding of LacR, and (3) minor decreases in association constant for a DNA-bound LacR molecule compared to free LacR that arise from greater translational and rotational entropy loss of the former in protein-DNA complexes [67].

Although an exact solution to the above system of equations is available and equivalent to solving a cubic equation, here we consider only the special case where *D_t_*<<*P_t_*, which corresponds to most *in vivo* conditions. In this case Equation (7) gives *p*_0_≈*P_t_*. The other variables can be obtained by replacing *p*_0_ with *P_t_*, expressing *d*_0_,*d*_1_,*d*_12_, and *d_c_* in terms of *d*_0_, and solving for *d*_0_ with Equation (8). Specifically,
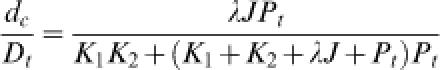
10


In the case where *λJ*>>*K*_1_, *K*_2_ and *λJ*>>*P_t_*, the looped configuration dominates and protein-DNA association can be approximated in terms of a two-state equilibrium, yielding
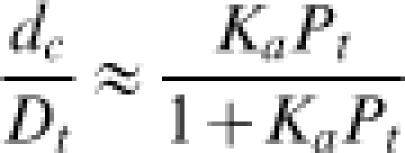
11


with an apparent association constant
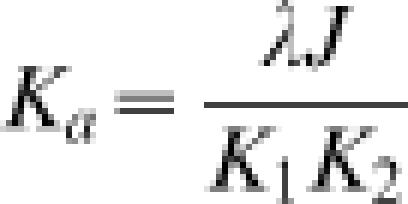
12


Equation (12) relates the total binding strength of a protein modeled as two DNA-binding domains connected by a flexible or semiflexible linker to the binding strengths of individual domains and the geometrical and mechanical properties of the linker. Although not formulated with J factors, a similar model was used by Crothers and Metzger to investigate the thermodynamic linkage between monomeric antibody binding strengths and the overall association constants of multivalent antibodies [68]. This model has been revisited in a study of protein-DNA interactions involving proteins with two domains connected by flexible linkers [69]. Here we have derived the general case from the standpoint of DNA looping and extended this approach to cases where the linkers are semi-flexible. The formula has recently been applied to quantitate the role of sequence-dependent DNA bending and flexibility in E2-DNA interactions using a worm-like chain model of DNA [31].

For the *lac* operon, we designate as “1” the primary site near the promoter, and “2” the auxiliary site located upstream of the promoter [5]. Based on the assumption that gene transcription is under thermodynamic control [4], the transcription rate of a reporter gene (the gene for β-galactosidase in this case) under control of the promoter is proportional to the probability that site “1” is free. Consequently, the gene transcription rate when DNA looping takes place is directly proportional to
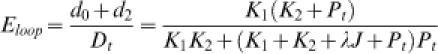
13When *P_t_*>>*K*_2_, Equation (13) can be simplified to
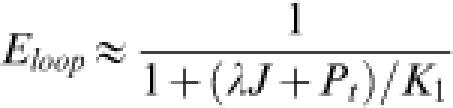
14which was previously obtained by Law et al [4], assuming 100% occupancy of site “2”. However, the relationship *P_t_*>>*K*_2_ does not hold in general and thus Equation (13) must be used. Similarly, the rate without DNA looping, which is determined in the absence of site “2”, is proportional to
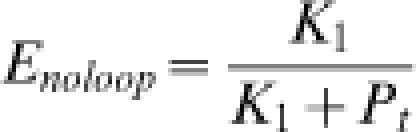
15


The enhanced gene repression due to DNA looping, *R*, can be expressed as the ratio of the specific enzymatic activity of β-galactosidase in the absence of the site “2” to that in its presence. Then the calculated enhanced gene repression, the ratio of transcription rates in the absence and presence of the loop, can be compared with the experimental *R* values through the relation shown in Equation 1.

## Supporting Information

Supporting Text Supporting information for “Analysis of *In-Vivo* LacR-Mediated Gene Repression Based on the Mechanics of DNA Looping” by Yongli Zhang, Abbye E. McEwen, Donald M. Crothers, and Stephen D. Levene.
(0.31 MB DOC)

Table S1 Comparison of calculated enhanced repression as a function of operator spacing with the *in-vivo* data of Müller *et al.* [4]
(0.27 MB DOC)

Table S2 Comparison of calculated enhanced repression as a function of operator spacing with the *in-vivo* data of Becker et al. [5] for wild-type (WT) and HU-deletion (*Δ*HU) *E. coli* strains.
(0.27 MB DOC)
