# Supplementary material for: Analysis of In-Vivo LacR-Mediated Gene Repression Based on the Mechanics of DNA Looping
Source: PLoS One. 2006 Dec 27;1(1):e136. doi: 10.1371/journal.pone.0000136 (PMC1762422; doi:10.1371/journal.pone.0000136)
Supplement: Supporting Text — Supporting information for “Analysis of In-Vivo LacR-Mediated Gene Repression Based on the Mechanics of DNA Looping” by Yongli Zhang, Abbye E. McEwen, Donald M. Crothers, and Stephen D. Levene. (0.31 MB DOC) [file pone.0000136.s001.doc]

# Analysis of *in-vivo* LacR-mediated Gene Repression Based on the Mechanics of DNA

# Looping

# Yongli Zhang, Abbye E. McEwen, Donald M. Crothers, and Stephen D. Levene

Supporting Information

Effects of LacR non-planarity on the J factor for the v-shaped tetramer conformation

The DNA helical axes in the LacR cocrystal structure with operator DNA do not lie in the mean plane of the tetramer subunits (Figure 1B), but instead are separated by a dihedral angle of about 20 degrees [1]. This implies that the crystallographic structure should introduce some writhe into a LacR-mediated loop, which could significantly affect the J factor. In particular, non-negligible writhe, depending on its sign, will couple differently with the (+) and (-) topoisomers of particular looped conformations. We examined the effect of LacR dimer/operator dihedral angle, which is equivalent to the protein-protein twist angle in our model,$\tau_{PP}$, for each of the three v-shaped conformations.

The strongest effects are generally observed for operator spacings that are torsionally out of phase. In these cases two loop topoisomers contribute to the J factor [2] and the differential coupling of $\tau_{PP}$ to the underwound, (-), and overwound, (+), topoisomers of particular LB (179-bp) and WA (163-bp) conformations can be clearly seen in Figures S1A and S1B, respectively. However, on taking the sum of (-) and (+) J-factor contributions, the overall effect is much less pronounced; there is less than 25% variation in $J$for the 179-bp LB loop and about a 5-fold parabolic dependence for the 163-bp WA loop over the range $-60^{^{\circ}}\leq\tau_{PP}\leq60^{^{\circ}}$ (the actual deviation for the non-planar WA 163 from the $\tau_{PP}=0$ value at the approximate crystallographic angle,$\tau_{PP}\approx+20^{^{\circ}}$, is about 50 percent). Non-planarity of the operators has negligible effects on LB and WA loops with optimal torsional phasing, in which cases single topoisomers dominate the J factor (Figures S1A, S1B). It is also noteworthy that the overall dependence of $J$ is quite symmetric with respect to the sign of$\tau_{PP}$.

We found a strong overall $\tau_{PP}$ dependence in the case of WT conformations (Figure S1C). Here, solutions almost exclusively involved a single topoisomer, probably owing to the nearly ring-like structure of WT loops, which suppresses writhe [3]. In fact, for the planar LacR structure, the increase in WT J factor with loop size (Figure 3A) indicates that the looping free energy is determined by elastic energy rather than entropy; thus, sensitivity to out-of-plane deformations is expected to be pronounced. The J factor for the 179-bp WT loop with nearly optimal torsional phasing increases by about a factor of 2 from$\tau_{PP}=0$ to$\tau_{PP}\approx+35^{^{\circ}}$, most likely reflecting increased loop entropy with the non-planar form of the LacR tetramer. At high absolute values of$\tau_{PP}$ J-factor values for sufficiently large WT loops approach those of the LB conformation. However, for loops smaller than 200 bp, all three v-shaped conformations still fail to compete thermodynamically with the extended SL conformation (Figure 3B).

**References**

1. Lewis M, Chang G, Horton NC, Kercher MA, Pace HC, et al. (1996) Crystal structure of the lactose operon repressor and its complexes with DNA and inducer. Science 271: 1247-1254.

2. Zhang Y, McEwen AE, Crothers DM, Levene SD (2006) Statistical-mechanical theory of DNA looping. Biophys J 90: 1903-1912.

3. Zhang YL, Crothers DM (2003) Statistical mechanics of sequence-dependent circular DNA and its application for DNA cyclization. Biophys J 84: 136-153.

4. Müller J, Oehler S, Müller-Hill B (1996) Repression of *lac* promoter as a function of distance, phase and quality of an auxiliary *lac* operator. J Mol Biol 257: 21-29.

5. Becker NA, Kahn JD, Maher LJ (2005) Bacterial repression loops require enhanced DNA flexibility. J Mol Biol 349: 716-730.
