# Supplementary material for: Analysis of In-Vivo LacR-Mediated Gene Repression Based on the Mechanics of DNA Looping
Source: PLoS One. 2006 Dec 27;1(1):e136. doi: 10.1371/journal.pone.0000136 (PMC1762422; doi:10.1371/journal.pone.0000136)
Supplement: Table S1 — Comparison of calculated enhanced repression as a function of operator spacing with the in-vivo data of Müller et al. [4] (0.27 MB DOC) [file pone.0000136.s002.doc]

| **Table S1**. Comparison of calculated enhanced repression as a function of operator spacing with the *in-vivo* data of Müller *et al.* [4]. | | | | | | | | |
| --- | --- | --- | --- | --- | --- | --- | --- | --- |
| Operator  spacing (bp) | Enhanced repression | | J factor  (nM) |  | Operator spacing  (bp) | Enhanced repression | | J factor  (nM) |
|  | Experimental | Calculated |  |  |  | Experimental | Calculated |  |
|  |  |  |  |  |  |  |  |  |
| 57.5 | 7.57 | 29.0 | 2391 |  | 87.5 | 10.4 | 3.49 | 212.5 |
| 58.5 | 14.6 | 31.5 | 2603 |  | 88.5 | 8.57 | 6.21 | 444.8 |
| 59.5 | 26.4 | 25.5 | 2094 |  | 89.5 | 9.86 | 12.3 | 964.2 |
| 60.5 | 8.29 | 15.8 | 1260 |  | 90.5 | 11.3 | 20.7 | 1681 |
| 61.5 | 10.9 | 7.73 | 575.0 |  | 91.5 | 32.3 | 28.8 | 2374 |
| 62.5 | 4.29 | 3.36 | 201.4 |  | 92.5 | 36.9 | 33.0 | 2730 |
| 64.5 | 3.29 | 2.39 | 118.5 |  | 93.5 | 23.1 | 31.1 | 2573 |
| 65.5 | 7.29 | 5.56 | 389.2 |  | 94.5 | 8.29 | 24.4 | 1999 |
| 66.5 | 7.29 | 13.3 | 1050 |  | 95.5 | 3.29 | 16.1 | 1287 |
| 67.5 | 8.86 | 25.9 | 2122 |  | 96.5 | 2.29 | 9.09 | 691.2 |
| 68.5 | 10.3 | 39.0 | 3245 |  | 97.5 | 3.43 | 4.98 | 340.1 |
| 69.5 | 15.3 | 45.4 | 3794 |  | 98.5 | 3.88 | 3.52 | 215.2 |
| 70.5 | 48.6 | 41.1 | 3424 |  | 111.5 | 3.14 | 5.32 | 368.9 |
| 71.5 | 24.3 | 29.2 | 2410 |  | 112.5 | 4.86 | 9.30 | 708.5 |
| 72.5 | 6.86 | 16.6 | 1334 |  | 113.5 | 6.14 | 14.4 | 1144 |
| 73.5 | 2.00 | 7.87 | 586.8 |  | 114.5 | 12.9 | 19.3 | 1559 |
| 74.5 | 2.71 | 3.62 | 223.9 |  | 115.5 | 29.3 | 22.1 | 1800 |
| 75.5 | 4.57 | 2.61 | 137.5 |  | 116.5 | 20.6 | 21.7 | 1769 |
| 76.5 | 5.14 | 4.37 | 287.4 |  | 117.5 | 11.0 | 18.4 | 1485 |
| 77.5 | 4.57 | 9.60 | 734.8 |  | 147.5 | 7.71 | 7.94 | 592.4 |
| 79.5 | 25.9 | 30.4 | 2507 |  | 149.5 | 7.43 | 13.0 | 1022 |
| 80.5 | 27.1 | 38.9 | 3237 |  | 150.5 | 12.9 | 14.0 | 1112 |
| 81.5 | 41.4 | 40.0 | 3326 |  | 151.5 | 7.29 | 13.5 | 1070 |
| 84.5 | 2.14 | 12.6 | 986.3 |  | 153.5 | 5.14 | 9.13 | 694.1 |
| 85.5 | 3.29 | 6.12 | 437.0 |  | 155.5 | 4.57 | 5.11 | 350.9 |
| 86.5 | 4.71 | 3.43 | 207.7 |  |  |  |  |  |
|  |  |  |  |  |  |  |  |  |

Preliminary fitting that included data points with operator spacing of 78.5 bp, 82.5 bp, and 83.5 bp yielded abnormally large fitting errors (≥3.0, see Table 1) and were therefore excluded from the final analysis. Average experimental uncertainty for a given enhanced-repression value is approximately 0.27 times the measured value.
