# Supplementary material for: Analysis of In-Vivo LacR-Mediated Gene Repression Based on the Mechanics of DNA Looping
Source: PLoS One. 2006 Dec 27;1(1):e136. doi: 10.1371/journal.pone.0000136 (PMC1762422; doi:10.1371/journal.pone.0000136)
Supplement: Table S2 — Comparison of calculated enhanced repression as a function of operator spacing with the in-vivo data of Becker et al. [5] for wild-type (WT) and HU-deletion (ΔHU) E. coli strains. (0.27 MB DOC) [file pone.0000136.s003.doc]

| **Table S2**. Comparison of calculated enhanced repression as a function of operator spacing with the *in-vivo* data of Becker *et al.* [5] for wild-type (WT) and HU-deletion (ΔHU) *E. coli* strains. | | | | | | | |
| --- | --- | --- | --- | --- | --- | --- | --- |
| Operator  spacing (bp) | Enhanced repression (WT) | | WT  J factor  (nM) |  | Enhanced repression (ΔHU) | | ΔHU  J factor (nM) |
|  | Experimental | Calculated |  |  | Experimental | Calculated |  |
|  |  |  |  |  |  |  |  |
| 63.5 | 19.0 | 35.0 | 729.4 |  | 10 | 8.3 | 157.8 |
| 64.5 | 37.1 | 63.2 | 1334 |  | - | - | 273.0 |
| 65.5 | 80.3 | 90.8 | 1926 |  | 9.8 | 18 | 355.9 |
| 66.5 | 104 | 105 | 2220 |  | 11 | 17 | 353.6 |
| 67.5 | 84.0 | 97.1 | 2061 |  | 11 | 14 | 271.1 |
| 70.5 | 18.4 | 23.9 | 492.2 |  | 3.0 | 2.6 | 33.48 |
| 71.5 | 17.9 | 14.0 | 279.4 |  | 3.0 | 2.4 | 29.27 |
| 72.5 | 15.8 | 14.6 | 291.4 |  | 5.4 | 4.1 | 66.64 |
| 73.5 | 8.57 | 26.1 | 537.8 |  | 8.5 | 8.7 | 164.7 |
| 74.5 | 11.2 | 51.3 | 1078 |  | 8.9 | 16 | 315.1 |
| 75.5 | 40.0 | 83.1 | 1760 |  | 6.5 | 23 | 470.8 |
| 76.5 | 129 | 111 | 2355 |  | 11 | 27 | 554.6 |
| 77.5 | 137 | 122 | 2603 |  | 12 | 25 | 519.6 |
| 78.5 | 125 | 113 | 2392 |  | 13 | 19 | 390.4 |
| 79.5 | 78.9 | 86.8 | 1840 |  | 9.1 | 12 | 237.2 |
| 80.5 | 53.5 | 56.6 | 1193 |  | 5.4 | 6.5 | 117.4 |
| 81.5 | 30.8 | 31.6 | 655.2 |  | 4.0 | 3.8 | 59.04 |
| 82.5 | 36.8 | 20.0 | 407.8 |  | 5.1 | 3.5 | 54.63 |
| 83.5 | 19.1 | 19.3 | 393.0 |  | 5.7 | 5.8 | 103.6 |
| 84.5 | 12.1 | 28.8 | 595.8 |  | 9.6 | 11 | 221.2 |
| 85.5 | 24.6 | 52.0 | 1094 |  | 21 | 19 | 378.6 |
| 86.5 | 83.0 | 79.4 | 1680 |  | 24 | 25 | 523.4 |
| 87.5 | 85.0 | 102 | 2171 |  | 30 | 28 | 588.5 |
| 88.5 | 87.3 | 112 | 2373 |  | 28 | 26 | 541.9 |
| 89.5 | 94.5 | 104 | 2206 |  | 26 | 20 | 411.3 |
| 90.5 | 59.3 | 82.8 | 1753 |  | 16 | 13 | 258.8 |
|  |  |  |  |  |  |  |  |

Average experimental uncertainty for particular enhanced-repression values are approximately 0.24 and 0.19 times measured values for wild-type and HU-deletion strains, respectively.
